# Changes of predominant species/biovars and sequence types of *Brucella* isolates, Inner Mongolia, China

**DOI:** 10.1186/1471-2334-13-514

**Published:** 2013-11-01

**Authors:** Yanfen Chen, Yuehua Ke, Yufei Wang, Xitong Yuan, Xiaoyan Zhou, Hai Jiang, Zhoujia Wang, Qing Zhen, Yaqin Yu, Liuyu Huang, Buyun Cui, Zeliang Chen

**Affiliations:** 1 Department of Infectious Disease Control, Beijing Institute of Disease Control and Prevention, Beijing 100071, P.R. China; 2 State Key Laboratory for Infectious Disease Prevention and Control, National Institute for Communicable Disease Control and Prevention, Beijing, P.R. China; 3 School of Public Health, Key Laboratory of Zoonosis, Ministry of Education, Jilin University, Changchun, Jilin 130021, P.R. China; 4 College of Medicine, Shihezi University, Shihezi 832003, P.R. China; 5 Research Center of Molecular Biology, Inner Mongolia Medical College, Hohhot, P.R. China

**Keywords:** *Brucella*, Genotype, Biovar, Multi locus sequence typing

## Abstract

**Background:**

Human brucellosis incidence in China was divided into 3 stages, high incidence (1950-1960s), decline (1970-1980s) and re-emergence (1990-2000s). Human brucellosis has been reported in all the 32 provinces, of which Inner Mongolia has the highest prevalence, accounting for over 40% of the cases in China. To investigate the etiology alteration of human brucellosis in Inner Mongolia, the species, biovars and genotypes of 60 *Brucella* isolates from this province were analyzed.

**Methods:**

Species and biovars of the *Brucella* strains isolated from outbreaks were determined based on classical identification procedures. Strains were genotyped by multi locus sequence typing (MLST). Sequences of 9 housekeeping genes were obtained and sequence types were defined. The distribution of species, biovars and sequence types (STs) among the three incidence stages were analyzed and compared.

**Results:**

The three stages of high incidence, decline and re-emergence were predominated by *B*. *melitensis* biovar 2 and 3, *B*. *abortus* biovar 3, and *B*. *melitensis* biovar 1, respectively, implying changes in the predominant biovars. Genotyping by MLST revealed a total of 14 STs. Nine STs (from ST28 to ST36), accounting for 64.3% of all the STs, were newly defined and different from those observed in other countries. Different STs were distributed among the three stages. ST8 was the most common ST in 1950-1960s and 1990-2000s, while ST2 was the most common in 1970-1980s.

**Conclusions:**

The prevalence of biovars and sequence types of *Brucella* strains from Inner Mongolia has changed over time in the three stages. Compared with those from other countries, new sequence types of *Brucella* strains exist in China.

## Background

Brucellosis is a zoonotic disease worldwide. It is classified as a reemerging infectious disease because of the increasing incidences in recent years. The genus *Brucella*, the etiological pathogen of brucellosis, is divided into at least ten species, including the six classical species: *B*. *abortus* (bovine), *B*. *melitensis* (caprine and ovine), *B*. *ovis* (ovine), *B*. *canis* (canine), *B*. *suis* (porcine), and *B*. *neotomae* on the basis of host specificity, antigenic differences and biochemical characteristics [1,2]. The classical species are divided into biovars even though the differences defining some biovars are minor and somewhat subjective. Four out of the six classical species are pathogenic for humans: *B*. *abortus*, *B*. *melitensis*, *B*. *suis* and rarely *B*. *canis*. *B*. *melitensis* and *B*. *abortus* are highly pathogenic and a frequent cause of human brucellosis.

Brucellosis is epidemic in China, and cases have been reported in all 32 provinces [3,4]. Since the 1950s, systematic surveys on the prevalent distributions and epidemiological characteristics of brucellosis have been carried out in China [4]. Human brucellosis was highly endemic from the mid-1950s well into the 1970s, and then incidence decreased until the mid-1990s, after which it increased sharply. Based on this change in incidences, brucellosis in China was divided into three periods, high incidence (1950-1960s), decline (1970-1980s) and re-emergence (1990-2000s) [4]. Although high incidence has been reported in many provinces, the etiological pathogen has not been genetically characterized. The main species, biovars, and genotypes of the *Brucella* isolates and their changes in the three different periods remain largely unknown. Of the epidemic foci, the Inner Mongolia Autonomous Region had the highest incidence, accounting for about 40% of the reported cases [5]. The incidence and epidemiology of brucellosis in this province could represent characteristics of brucellosis in China [4]. Spatial and temporal distributions of human brucellosis in this province during the time period of 1999–2008 were systematically investigated in a recent study. The annual incidence varied greatly from 0 to 818.52/100,000 at county levels during the study period. The highest incidence of the disease occurred in Abaga County. The spatial distribution of the disease clustered in the northeastern and central districts [5].

Multi locus sequence typing (MLST) is a DNA sequence-based typing method for many different bacterial species to differentiate strains and identify clonal lineages [6]. This technique is appropriate for global or long-term epidemiology and surveillance, and has the advantages over other typing methods, such as genetic fingerprinting of electronic portability and unambiguous characterization of isolates. MLST data are easily stored in databases that can be exchanged between different laboratories [7]. MLST has also been applied to genetic analysis of 160 strains of different *Brucella* species and biovars [8]. A total of 27 sequence types were defined among these strains. An extended MLST method was also developed for *Brucella*, with which the *Brucella* isolates could be differentiated with higher resolution [9].

To investigate etiological changes of brucellosis in China, in the present study, species, biovars and genotypes of *Brucella* isolates mainly from outbreaks in Inner Mongolia were analyzed and compared. The most common biovars and genotypes during the three periods were investigated.

## Methods

### Sample collection

All *Brucella* isolates were collected and preserved by the State Key Laboratory for Infectious Disease Prevention and Control, National Institute for Communicable Disease Control and Prevention. *Brucella* field strains were isolated from clinical samples from human Brucellosis outbreaks. The samples were collected as part of standard patient care between 1955 and 2008 and were fully de-identified.

### Bacterial strains

All isolates were identified as *Brucella* species and biovar on the basis of classical identification procedures: CO2 requirement, H2S production, inhibition of growth by basic fuchsin and thionin, agglutination with monospecific antisera and phage typing. All the isolates used for the present study were preserved without extensive laboratory passage. *Brucella* strains were grown to stationary phase at 37°C in Tryptic Soy Broth (TSB) or Tryptic Soy Agar (TSA) with or without 5% CO_2_. Genomic DNA was isolated from *Brucella* cultures by using a Wizard SV Genomic DNA purification kit.

### PCR amplification and sequencing

Nine distinct genomic loci, including seven housekeeping genes, one outer membrane protein, and one intergenic fragment, were selected as described previously [8]. PCR reaction mixes were prepared by mixing 5 μl 10× PCR Buffer with MgCl_2_, 4 μl 2.5 mM dNTPs, 1 μl of each primer (20 μM), 0.25 μl of Pyrobest DNA polymerase (5 U/μl), 33.75 μl of distilled water and 5 μl of diluted genomic DNA (10 ng). Cycling parameters were as follows: 94°C for 5 min, followed by 30 cycles of 94°C for 0.5 min, 63°C for 0.5 min, and 72°C for 1 min, and at last an elongation step of 72°C for 10 min. Products were separated by agarose gel electrophoresis to check for success of amplification and to ensure that only a single product of the expected size was present. PCR products were then purified and sequenced with ABI 3730 with Big Dye terminator cycle sequencing kit version 3.1 (Applied Biosystems) at the Beijing Genomics Institute.

### Sequence analysis

The raw sequence data were edited using Edit module and contigs were generated from forword and reverse sequences by using SeqMan module of the Lasergene package (version 5) [10]. MLST sequences (accession number from AM694191 through AM695630) of the strains described by Adrian M Whatmore were downloaded from GeneBank database. Each distinct allele at each of the nine loci was given a numerical designation according to sequence of defined alleles. If the sequence is different from those defined previously, a new allele was defined. Each unique allelic pattern over all loci was identified as a sequence type (ST). Allelic profiles and sequence data were imported into the START package to determine mean GC content [11]. The same package was used to calculate the average frequencies of synonymous substitutions per potential synonymous site (d_S_) and nonsynonymous substitutions per potential nonsynonymous site (d_N_) by the method of Nei and Gojobori in order to test the degree of selection on a locus. A representative strain of each genotype (ST) was used for phylogenetic analysis. Sequences of the nine loci were then concatenated and used to infer phylogentic relationships of the representative strains. Phylogenetic analyses were conducted in with MEGA 5 [12]. The evolutionary relationships were inferred using the Neighbor-Joining method [13]. A bootstrap consensus tree was generated to assess the phylogenetic support of the branching [14]. Split decomposition analysis of allelic profile data was performed using a web-based version of the SplitsTree program [15].

## Results

### Different prominent species and biovars during three incidence stages

Human brucellosis has been systematically surveyed in China since it was confirmed in the 1950s. According to the epidemiological data obtained from these surveys, brucellosis was highly prevalent in the 1950s and 1960s, and then decreased in the 1970s and 1980s period. However, since the 1990s, the incidence increased sharply, forming clear three stages: high incidence (1950-1960s), decline (1970-1980s) and re-emergence (1990-2000s) [4]. Therefore, the species and biovars of the isolates were analyzed and compared between the three stages. A total of 60 strains were isolated in Inner Mongolia from 1955 to 2008, including 24 *B*. *abortus*, 34 *B*. *melitensis* and 2 *B*. *suis*. In total, 18, 21 and 21 strains were isolated in 1950-1960s, 1970-1980s and 1990-2000s stages, respectively (Table 1). Species and biovars of these isolates were analyzed and compared according to the three incidence stages. As shown in Table 2, in 1950-1060 s, 3 *B*. *abortus* (1 biovar 1 and 3 biovar 3), 13 *B*. *melitensis* (6 biovar 2 and 7 biovar 3) and 2 *B*. *suis* (1 biovar 1 and 1 biovar 3) were isolated, and in 1970-1980s, 17 *B*. *abortus* (4 biovar 1, 11 biovar 3 and 2 biovar 6) and 4 *B*. *melitensis* (4 biovar 1) were isolated, while in 1990-200 s, 4 *B*. *abortus* (4 biovar 3) and 17 *B*. *melitensis* (17 biovar 1) were isolated. In the high incidence stages of 1950-1960s and 1990-2000s, the most common species was *B*. *melitensis*. In the low incidence stage of 1970-1980s, the most common species was *B*. *abortus*. Furthermore, the main biovars differ between the two high incidence stages, with *B*. *melitensis* biovar 2 and biovar 3 in 1950-1960s and *B*. *melitensis* biovar 1 in 1990-2000s respectively. Distribution difference analysis by Chi-square test showed that species were significantly differentially distributed among the three stages (*χ*^2^ = 26.425, p < 0.001).

**Table 1:** **Species**, **biovar**, **sequence type**, **clonal complex and isolation time of the 60 isolates from Inner Mongolia**

**Strains**	**ST**^**a**^	**Time**	**Period**	**Stage**^**b**^	**Species**	**Biovar**^**c**^
Bru001	8	1955	1950s	1950-1960s	*B*. *melitensis*	2
Bru002	8	1958	1950s	1950-1960s	*B*. *melitensis*	2
Bru003	8	1960	1960s	1950-1960s	*B*. *melitensis*	2
Bru004	34	1963	1960s	1950-1960s	*B*. *melitensis*	2
Bru005	8	1965	1960s	1950-1960s	*B*. *melitensis*	2
Bru006	34	1966	1960s	1950-1960s	*B*. *melitensis*	2
Bru007	8	1957	1950s	1950-1960s	*B*. *melitensis*	3
Bru008	8	1957	1950s	1950-1960s	*B*. *melitensis*	3
Bru009	8	1957	1950s	1950-1960s	*B*. *melitensis*	3
Bru010	8	1958	1950s	1950-1960s	*B*. *melitensis*	3
Bru011	8	1958	1950s	1950-1960s	*B*. *melitensis*	3
Bru012	8	1960	1960s	1950-1960s	*B*. *melitensis*	3
Bru013	8	1965	1960s	1950-1960s	*B*. *melitensis*	3
Bru014	2	1960	1960s	1950-1960s	*B*. *abortus*	1
Bru018	5	1956	1950s	1950-1960s	*B*. *abortus*	3
Bru019	2	1956	1950s	1950-1960s	*B*. *abortus*	3
Bru022	36	1957	1950s	1950-1960s	*B*. *suis*	1
Bru023	17	1963	1960s	1950-1960s	*B*. *suis*	3
Bru015	28	1972	1970s	1970-1980s	*B*. *abortus*	1
Bru016	1	1980	1980s	1970-1980s	*B*. *abortus*	1
Bru020	33	1979	1970s	1970-1980s	*B*. *abortus*	6
Bru021	31	1980	1980s	1970-1980s	*B*. *abortus*	6
Bru024	2	1988	1980s	1970-1980s	*B*. *abortus*	3
Bru025	2	1988	1980s	1970-1980s	*B*. *abortus*	3
Bru026	1	1988	1980s	1970-1980s	*B*. *abortus*	1
Bru027	29	1989	1980s	1970-1980s	*B*. *abortus*	3
Bru028	29	1985	1980s	1970-1980s	*B*. *abortus*	3
Bru029	32	1985	1980s	1970-1980s	*B*. *abortus*	3
Bru030	8	1985	1980s	1970-1980s	*B*. *melitensis*	1
Bru031	2	1985	1980s	1970-1980s	*B*. *abortus*	3
Bru032	30	1985	1980s	1970-1980s	*B*. *abortus*	1
Bru033	29	1988	1980s	1970-1980s	*B*. *abortus*	3
Bru034	2	1988	1980s	1970-1980s	*B*. *abortus*	3
Bru035	2	1988	1980s	1970-1980s	*B*. *abortus*	3
Bru036	2	1988	1980s	1970-1980s	*B*. *abortus*	3
Bru037	29	1988	1980s	1970-1980s	*B*. *abortus*	3
Bru038	8	1988	1980s	1970-1980s	*B*. *melitensis*	1
Bru039	34	1989	1980s	1970-1980s	*B*. *melitensis*	1
Bru040	8	1989	1980s	1970-1980s	*B*. *melitensis*	1
Bru017	1	2006	2000s	1990-2000s	*B*. *abortus*	1
Bru041	34	1993	1990s	1990-2000s	*B*. *melitensis*	1
Bru042	8	1990	1990s	1990-2000s	*B*. *melitensis*	1
Bru043	34	2001	2000s	1990-2000s	*B*. *melitensis*	1
Bru044	8	1996	1990s	1990-2000s	*B*. *melitensis*	1
Bru045	8	1997	1990s	1990-2000s	*B*. *melitensis*	1
Bru046	8	1998	1990s	1990-2000s	*B*. *melitensis*	1
Bru047	8	1996	1990s	1990-2000s	*B*. *melitensis*	1
Bru048	8	1999	1990s	1990-2000s	*B*. *melitensis*	1
Bru049	34	2008	2000s	1990-2000s	*B*. *melitensis*	1
Bru050	8	1998	1990s	1990-2000s	*B*. *melitensis*	1
Bru051	8	2002	2000s	1990-2000s	*B*. *melitensis*	1
Bru052	34	2002	2000s	1990-2000s	*B*. *melitensis*	1
Bru053	35	1997	1990s	1990-2000s	*B*. *melitensis*	1
Bru054	34	2007	2000s	1990-2000s	*B*. *melitensis*	1
Bru055	34	1996	1990s	1990-2000s	*B*. *melitensis*	1
Bru056	8	2007	2000s	1990-2000s	*B*. *melitensis*	1
Bru057	8	2006	2000s	1990-2000s	*B*. *melitensis*	1
Bru058	2	1995	1990s	1990-2000s	*B*. *abortus*	3
Bru059	29	1996	1990s	1990-2000s	*B*. *abortus*	3
Bru060	2	1997	1990s	1990-2000s	*B*. *abortus*	3

^a^ST represent sequence type. ^b^Stage represent the incidence stages: high incidence of 1950-1960s, low incidence stage 1970-1980s, and re-emerging high incidence stage of 1990-2000s. ^c^The Arabic number represents the biovar of the species.

**Table 2:** Species and biovar distribution during the three incidence stages

**Species/Biovars**		**Incidence stage**^**a**^			**Total**
**Species**	**Biovars**	**1950-1960s**	**1970-1980s**	**1990-2000s**	
*B*. *melitensis*	1	0	4	17	21
	2	6	0	0	6
	3	7	0	0	7
*B*. *abortus*	1	1	4	1	6
	3	2	11	3	16
	6	0	2	0	2
*B*. *suis*	1	1	0	0	1
	3	1	0	0	1
Total		18	21	21	60

^a^The number represent the strain number of respective incidence stage.

### Different sequence type distributions among the three incidence stages

To analyze the genetic polymorphism of these isolates by MLST, nine distinct genomic loci of the 60 strains were sequenced and their sequences analyzed. Each distinct allele at each locus was given a numerical designation and each unique allelic pattern over all nine loci was defined as a sequence type (ST) according to a previous study [8]. A total of 14 distinct STs were defined. The most common sequence type is ST8 (24 strains, 40.0%), followed by ST2 (10 strains, 16.7%), ST34 (9 strains, 15.0%), ST29 (5 strains, 8.3%) and ST1 (3 strains, 5.0%). The other 9 STs were represented by only 1 strain each (Table 3). ST8 is the most common sequence type in 1950-1960s and 1990-2000s, accounting up to 61.1% and 47.6% of the isolates of the stages. However, ST8 was observed in only 14.29% of the isolates in 1970-1980s. Analysis with Chi-square showed that the distribution difference of ST8 among the three stages was significant (*χ*^2^ = 9.636, p = 0.006). In 1970-1980s, the most represented sequence type was ST2 (6 strains, 28.57%). The number of sequence type differed among the three stages, but without significance (*χ*^2^ = 3.314, p = 0.247). Eighteen strains from the stage of 1950-1960s belonged to 6 STs (3 strains per ST), and 21 strains from 1990-2000s belonged to 6 STs (3.5 strains per ST). However, the 21 strains from 1970-1980s belonged to 10 STs (2.1 strains per ST). When analyzed in general, these STs were differentially distributed among the three period with significance (*χ*^2^ = 37.390, p = 0.007). Compared with sequence types of strains from other countries, 9 of the 14 sequence types (from ST28 to ST36) were first observed by this study. This implied that sequence types of strain from Inner Mongolia were greatly different from those from other countries.

**Table 3:** Sequence type distributions during the three incidence stages

**ST**	**Incidence stage**^**a**^			**Total**
	**1950-1960s**	**1970-1980s**	**1990-2000s**	
1		2 (9.52%)	1 (4.76%)	3 (5.00%)
2	2 (11.11%)	6 (28.57%)	2 (9.52%)	10 (16.67%)
5	1 (5.56%)			1 (1.67%)
8	11 (61.11%)	3 (14.29%)	10 (47.62%)	24 (40.00%)
17	1 (5.56%)			1 (1.67%)
28		1 (4.76%)		1 (1.67%)
29		4 (19.05%)	1 (4.76%)	5 (8.33%)
30		1 (4.76%)		1 (1.67%)
31		1 (4.76%)		1 (1.67%)
32		1 (4.76%)		1 (1.67%)
33		1 (4.76%)		1 (1.67%)
34	2 (11.11%)	1 (4.76%)	6 (28.57%)	9 (15.00%)
35			1 (4.76%)	1 (1.67%)
36	1 (5.56%)			1 (1.67%)

^a^The number represent strain number. The percentage in brackets represents the percentage of the strains in the time period.

### Sequence polymorphism and alleles changes of the nine loci for predominant species during the three incidence stages

The polymorphic alleles and sites were analyzed for the nine genes. The number of alleles for nine genes ranged from 2 (*dna*K) up to 6 (*aro*A). The number of polymorphic sites ranged from 1 (*dna*K) up to 4 (*omp*25). The dN/dS ratio was calculated to determine the degree of selection in the sequence population. The dN/dS ratio ranged from 0 to 0.7678. The dN/dS ratio of *dna*K and *omp*25 was 0. *GlK* showed the highest dN/dS ratio of 0.761. The GC% content for the eight genes ranges from 55.8% to 62.5%, with an average of 59.4%, being higher than average (57.0%) of the whole genome sequence (Table 4).

**Table 4:** Sequence polymorphism of the 9 loci

**Locus**	**Alleles**	**Polymorphic sites**	**Polymorphic sites percent**	**d**_**N**_	**d**_**S**_	**d**_**N**_**/d**_**S**_	**Mean % GC**
*aroA*	6	4	0.71%	0.0026	0.0058	0.4422	61.77
*cobQ*	5	4	0.95%	0.0044	0.0058	0.7678	59.43
*dnak*	2	1	0.21%	0	0.009	0	60.53
*gap*	3	3	0.51%	0.0015	0.0091	0.1668	58.35
*glk*	4	4	0.84%	0.0029	0.0038	0.761	62.53
*gyrB*	5	3	0.64%	0.0017	0.0102	0.1681	58.93
*omp25*	5	4	0.82%	0	0.02	0	59.2
*trpE*	3	2	0.41%	0.0018	0.0056	0.3239	58.44
*Int*-*hyp*	3	4	0.93%	-	-	-	55.81

To further analyze the polymorphism of the predominant species, allele changes in nine loci of the sequence type were compared (Table 5). In 1950-1960s, the most represented species was *B*. *melitensis*, and the sequence type is ST8 and ST34. Compared with ST8, only one site was changed in ST34. Allele 8 of omp25 was changed to allele 12. In 1990-2000s stages, the sequence types are still most represented by ST8 and ST34, but a new sequence type ST35 was observed. Compared with ST34, the allele of *gyr*B was changed from 1 to 8. However, only 1 strain of ST35 was observed. *B*. *abortus* is the predominant species at low incidence stages. In 1950-1960s, only 3 *B*. *abortus* strains were isolated, they belonged to 2 different STs, ST2 and ST5. 2 of the 9 loci showed allele changes between the 2 STs. Alleles of *gl*K were changed from 2 to 1, and *trp*E from 3 to 4. In 1970-1980s, 8 STs were observed for *B*. *abortus*. The most represented sequence type was ST2 (6 strains) and ST29 (4 strains). The two sequence types had only 1 allele differences. The allele of *omp*25 was changed from 1 in ST2 to 11 in ST9. For all the 8 STs observed in *B*. *abortus* strains from 1970-1980s, 6 of the 9 loci changed. A total of 19 alleles for all the loci were observed, indicating the high diversity of *B*. *abortus* in this incidence stages. Compared with alleles of the nine loci from strains of other countries, new alleles were observed in four loci, including aroA (allele 8, 9 and 10), cobQ (allele 8, 9), gyrB (allele 7), omp25 (allele 11, 12).

**Table 5:** Allele profile and strains distribution of the 14 sequence types

**ST**^**a**^	**Species**	**Allele distribution**^**b**^								
		***aro*A**	***cob*Q**	***dna*k**	***ga*p**	***gl*k**	***gyr*B**	***omp*25**	***trp*E**	***Int-hyp***
1	*B. abortus*	1	1	2	2	1	1	1	3	1
2	*B. abortus*	1	1	2	2	2	1	1	3	1
5	*B. abortus*	1	1	2	2	1	1	1	4	1
28	*B. abortus*	1	1	2	2	1	1	11	4	1
29	*B. abortus*	1	1	2	2	2	1	11	3	1
30	*B. abortus*	1	1	2	2	3	1	1	3	1
32	*B. abortus*	8	1	2	2	2	1	1	3	1
31	*B. abortus*	1	8	2	2	1	1	1	3	2
33	*B. abortus*	10	9	2	2	4	7	1	3	1
8	*B. melitensis*	2	3	2	3	3	1	8	5	2
34	*B. melitensis*	2	3	2	3	3	1	12	5	2
35	*B. melitensis*	2	3	2	3	3	8	12	5	2
17	*B. suis*	6	5	1	1	4	5	2	3	4
36	*B. suis*	9	5	1	1	4	4	2	3	1

^a^ST represents sequence type; ^b^Alleles were defined based on previous definition.

### Combined comparison of sequence type and species-biovar changes during the three incidence stages

In 1950-1960s, 2 biovars of *B*. *melitensis*, biovar 2 and 3, were identified. The 2 biovars belonged to 2 sequence types, ST8 and ST34. However, there was not a strict consistency between the STs and biovars. Both strains of *B*. *melitensis* biovar 2 and biovar 3 belonged to ST8 and ST34. For species *B*. *abortus*, 2 biovars and STs were observed. In 1970-1980s, for the most commnon species *B*. *abortus*, 3 biovars (1, 3 and 6) and 8 STs were observed. The most common sequence type and biovar were ST2 and biovar 3. Interestingly, strains of the most represented sequence type ST2 and ST29 belonged to biovar 3. Therefore, in this low incidence stage, biovar 3 and sequence type ST2 and ST29 are the most distributed. In 1990-2000s, *B*. *melitensis* was the most abundant species, and only one biovar (biovar 1) was identified. These strains belonged to 3 STs, mostly represented by ST8 and ST34, sequence type with only 1 allele different. Compared with those in 1950-1960s, sequence type and biovar of *B*. *melitensis* in 1990-2000s showed an interesting change. Although still most represented by ST8, more ST34 strains observed in 1990-2000s. Biovar 1 in 1990-2000s replaced biovar 2 and 3 in 1950-1960s.

### Phylogenetic relationship *Brucella* isolates

**Figure 1:**
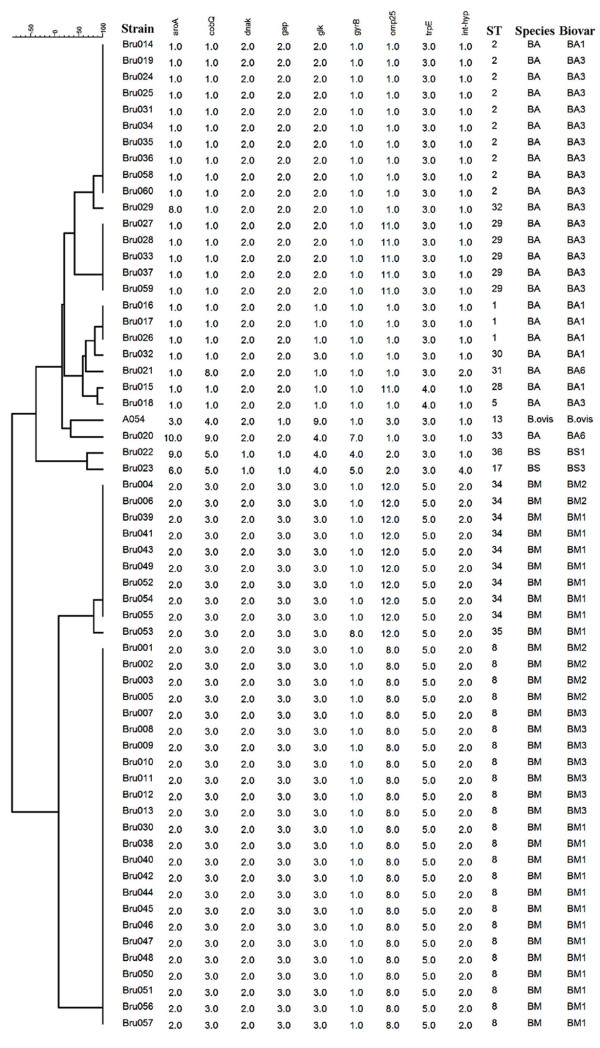
**Phylogeny based on concatenated sequence of 9 loci showing the relationships of the 60 isolates.** Sequences of each sequence type were concatenated and phylogenetic trees were analyzed by using neighbor-joining method. *B*. *ovis* was used as an outgroup.

To further test the phylogenetic structure of the isolates, sequences of the 9 loci for each of the strains were concatenated and analyzed in MEGA 5. All the strains clustered into 15 branches, representing a total of 15 sequence types. As shown in Figure 1, these strains formed two clades. The 2 *B*. *suis* isolates form a clade.

## Discussions

Human brucellosis in China has been documented from the early 1900s. The earliest two cases of brucellosis in China were reported to occurred in Shanghai by Boone in 1905 [16]. The two cases were diagnosed as having Malta fever by clinical symptoms and limited epidemiological data. In 1925, Y. Zongyang reported four cases [4]. Antibodies were detected and *B*. *melitensis* was isolated from one of the four patients. This represented the first confirmed brucellosis cases in China. Although systematic surveys and effective measures were not taken for the prevention and control of brucellosis before 1950, the published data suggested that both human and animal brucellosis were present in many provinces of China before 1950 [4]. Since 1950, systematic surveys of both human and animal brucellosis have been carried out. From 1952 to 1990, surveys involving about 79.7 million animals of more than 20 species of domestic and wild animals showed a positive rate of 17.3% for animal brucellosis. Human brucellosis positive rate during this period was about 3.56% [17]. The general epidemiological characteristics of brucellosis in China are as follows: in 1950-1960s, human and animal brucellosis was quite severe; in 1970-1980s, the incidence of human and animal brucellosis was relatively low, and seemed to decrease during this period; in 1990-2000s, there were no obvious change of animal brucellosis, but the incidence of human brucellosis increased. Because humans are infected by *Brucella* through contact with infected animals, the increase or decrease of human brucellosis rate usually follows the same trend of animal brucellosis [18]. The main reason for contrast in incidence of human and animal brucellosis was considered to be resulted from the differences in the time, place, tests and sampling methods of the surveys on human and animal brucellosis [18].

Etiological analysis of human and animal brucellosis revealed prominent *Brucella* species in China [18]. Analysis of the *Brucella* strains isolated from human and animals showed that the most common species was *B*. *melitensis*, followed by *B*. *abortus* and *B*. *suis*. Only a small proportion of the cases are caused by *B*. *ovis* and atypical *Brucella* strains [3]. *B*. *melitensis* was the most prominent species associated with outbreaks. But the prominent species differed among provinces [17]. For the regions where at least two species exist, the predominant strains were *B*. *melitensis* with either *B*. *abortus* and/or *B*. *suis*[4].

Inner Mongolia has experienced a high incidence in 1950-1960s, a decrease in 1970-1980s and re-emergence of high incidence since 1990s [4]. Because most of the historical isolates were not preserved, only limited number of strains could be included in this study. Since most of these strains were isolated from brucellosis outbreaks, it is possible to investigate general trends in terms of species/biovar and genotype changes. In 1950-1960s and 1990-2000s, when the incidence was high, a higher percentage of *B*. *melitensis* was observed. In 1970-1980s, more *B*. *abortus* were isolated.

In 1950-1960s, 3 species and 6 biovars of *Brucella* were isolated. In 1970-1980s, 2 species (*B*. *abortus* and *B*. *melitensis*) and 5 biovars were isolated. In 1990-2000s, 2 species (*B*. *abortus* and *B*. *melitensis*) but only 3 biovars were isolated. This indicated that in Inner Mongolia, diversity in epidemic species and biovars was reduced during the three stages. At high incidence stages, *B*. *melitensis* was the most represented species. But the epidemic biovars for the two stages differed. In 1950-1960s, 6 biovar 2 and 7 biovar 3 strains were isolated. But in 1990-2000s, all 17 *B*. *melitensis* strains were biovar 1. That is, the main biovars of *B*. *melitensis* have changed in the two time periods. Previous epidemiological data showed that *B*. *melitensis* was predominant species that was associated with outbreaks throughout the country [17]. Results from the present study further implied that different biovars of this species were associated with the high incidence and outbreaks for the two stages.

Sequence type analysis revealed a total of 14 STs. Most of the STs were less common. Nine STs contained only one strain. The most represented ST is ST8, which contained 24 strains, accounting for 40.1% of all the tested strains. Comparison of ST distributions between the three stages showed that ST8 is the most common STs in 1950-1960s and 1990-2000s, two high incidence stages. ST2 is the most common sequence type during the low incidence stage 1970-1980s. All the ST2 strains belonged to species *B*. *abortus*. ST distribution analysis showed that 6 STs were observed at the two high incidence stages, but 10 STs were observed at the low incidence stage. The reason for the diversity differences between sequence types of the strains might be the differences in species and biovars distributions. During the high incidence stage, *B*. *melitensis* was the most common species, while in low incidence stage, *B*. *abortus* was the most represented species. Further analysis of the isolates showed that the number of intraspecies polymorphisms differed among the three species. *B*. *abortus* was the most genetic diverse species. A total of 20 polymorphic sites were observed in sequences of the 24 *B*. *abortus* strains. Nine STs and biovar 1, 3 and 6 were observed for these *B*. *abortus* strains. *B*. *melitensis* was more homogenous, 3 STs and 3 biovars were observed. No clear relationship between biovar and ST within both *B*. *melitensis* and *B*. *abortus* was observed. This was in agreement with previous MLST results. The inconsistence of genotype and biovars has been observed in analysis with other typing approaches [2].

MLST is a molecular genotyping method appropriate for global analysis. We also compared sequence types of strain from Inner Mongolia and those from other countries. Of the 14 STs, 9 (64.28%) were firstly observed in strains from Inner Mongolia. The ST differences were resulted from differences in alleles. New alleles were observed in 4 of the 9 loci. These data implied that *Brucella* strains from Inner Mongolia were greatly different from those from other countries.

Generally, changes of genotype are not completely consistent with that of species or biovars of bacterial strains [8,19]. Combined comparison between sequence type and species-biovar changes during the three incidence stages showed that data from this study was also consistent with that. For the most represented species *B*. *melitensis*, in 1950-1960s, biovar 2 and 3 were mostly represented. These strains belonged to ST8 and ST34. And in 1990-2000s, biovar 1 wasmost common, the sequence types were still ST8 and ST34, but more ST34 appeared. That is, the biovars has been changed greatly, but the genotype remained unchanged.

## Conclusions

In summary, in the present study, we analyzed species, biovars and sequence types of 60 *Brucella* stains isolated from different incidence stages in Inner Mongolia, the province in China with highest incidence of brucellosis. The species and biovars of the isolates from the high incidence stage were significantly different from that from low incidence stage. Genotyping by MLST of the isolates showed that sequence types of strains from high incidence stages differed from those from the low incidence stage. Both the biovars and genotypes in 1990-2000s were different from that in 1950-1960s, indicated the emergence of new biovars and genotypes of *Brucella* strains. This implied that the etiology alterations might be a reason for brucellosis incidence changes in China. Further epidemiological investigation will be greatly helpful for explanation of the etiology changes of brucellosis in China.

## Abbreviations

MLST: Multi locus sequence typing; ST: Sequence type.

## Competing interests

The authors declare that they have no competing interests.

## Authors’ contributions

YC, YK, YW, XY, XZ, HJ performed the study and analyzed the data, ZW, QZ, YY, LH, BC, ZC conceived the study and analyzed the data, YC, YK, YW, ZC drafted and edited the manuscript. All authors read and approved the final manuscript.

## Pre-publication history

The pre-publication history for this paper can be accessed here:

http://www.biomedcentral.com/1471-2334/13/514/prepub
